# Purification, Characterization and Degradation Performance of a Novel Dextranase from *Penicillium cyclopium* CICC-4022

**DOI:** 10.3390/ijms20061360

**Published:** 2019-03-18

**Authors:** Ruijie Huang, Lei Zhong, Fengwei Xie, Liming Wei, Lanfang Gan, Xuejiao Wang, Anping Liao

**Affiliations:** 1 Guangxi Key Laboratory for Polysaccharide Materials and Modifications, School of Chemistry and Chemical Engineering, Guangxi University for Nationalities, Nanning 530006, China; 18238610490@163.com (R.H.); leiwin@gmail.com (L.Z.); 15177897741@163.com (L.W.); 15177519070@163.com (L.G.); wxj2602026707@163.com (X.W.); 2 International Institute for Nanocomposites Manufacturing (IINM), WMG, University of Warwick, Coventry CV4 7AL, UK; D.Xie.2@warwick.ac.uk; 3 School of Chemical Engineering, The University of Queensland, Brisbane, Qld 4072, Australia

**Keywords:** *Penicillium cyclopium*, dextranase, purification, molecular mass, degradation, dextran

## Abstract

A novel dextranase was purified from *Penicillium cyclopium* CICC-4022 by ammonium sulfate fractional precipitation and gel filtration chromatography. The effects of temperature, pH and some metal ions and chemicals on dextranase activity were investigated. Subsequently, the dextranase was used to produce dextran with specific molecular mass. Weight-average molecular mass (*M*_w_) and the ratio of weight-average molecular mass/number-average molecular mass, or polydispersity index (*M*_w_/*M*_n_), of dextran were measured by multiple-angle laser light scattering (MALS) combined with gel permeation chromatography (GPC). The dextranase was purified to 16.09-fold concentration; the recovery rate was 29.17%; and the specific activity reached 350.29 U/mg. *M*_w_ of the dextranase was 66 kDa, which is similar to dextranase obtained from other *Penicillium* species reported previously. The highest activity was observed at 55 °C and a pH of 5.0. This dextranase was identified as an endodextranase, which specifically degraded the α-1,6 glucosidic bonds of dextran. According to metal ion dependency tests, Li^+^, Na^+^ and Fe^2+^ were observed to effectively improve the enzymatic activity. In particular, Li^+^ could improve the activity to 116.28%. Furthermore, the dextranase was efficient at degrading dextran and the degradation rate can be well controlled by the dextranase activity, substrate concentration and reaction time. Thus, our results demonstrate the high potential of this dextranase from *Penicillium cyclopium* CICC-4022 as an efficient enzyme to produce specific clinical dextrans.

## 1. Introduction

Dextranase (1,6-α-d-glucan-6-glucanohydrolase; E.C. 3.2.1.11) is an inducible enzyme and can specially catalyse the endohydrolysis of α-(1,6)-d-glycoside linkages at random sites of dextran. The main products are isomaltose, isomaltotriose, small amounts of d-glucose and a series of low molecular-mass polysaccharides [1]. Dextranase has been widely used in the production of specific clinical dextrans [2,3]. Dextran molecules produced by dextranase with relatively low weight-average molecular mass (*M*_w_) of 20–70 kDa can be used as blood extenders and those with *M*_w_ of 6–8 kDa can form complexes with iron to treat severe anaemia [4]. Since dextranase can effectively degrade dextran in dental plaques, the use of this enzyme has become an effective way to treat dental caries [5,6,7,8].

Currently, bacteria and fungi are two of the main sources to produce dextranases [9]. Dextranases from bacteria usually have favorable thermal stability but relatively low dextranase activity. Hide et al. [10] obtained a dextranase from *Paenibacillus* sp. mutant with the optimal temperature of 60 °C. Rashida et al. [11] purified a dextranase from *Bacillus licheniformis* KIBGE-IB25, of which the specific activity was 1405 U/mg. Compared with dextranases from bacteria, those from fungi may achieve higher activities. Wu et al. [4] obtained a dextranase from *Hypocrea lixii* F1002, of which the specific enzyme activity was 2782 U/mg. Siwames et al. [12] acquired a dextranase from *Aspergillus allahabadii* X26, of which the specific activity reached 3009 U/mg. Dextranases from *Penicillium* have been found to have high dextranase activity as well as excellent temperature and pH stability. Zhang et al. [13] screened *Talaromyces pinophilus* H6 from the soil, which was found to be stable at 35–60 °C and pH of 3.0–10.0. This special dextranase displayed activity as high as 14,894 U/mg. 

Previous research about dextranases has mostly focused on the mutation [9], screening [14], construction of genetically engineered bacteria [15,16], the optimization of fermentation medium [17] and fermentation conditions [18] and the purification and enzymatic properties [19,20,21]. To the best of our knowledge, there are few studies on how to regulate molecular mass of dextran during production. Since the molecular mass of dextrans largely determines their applications, it is essential to find an efficient way to control this parameter. Besides, we found that the determination of the molecular mass of dextrans was inaccurate in most reports [4,13]. In these reports, weight-average molecular weight (*M*_w_) was detected and determined by a refractive index detector and its accuracy relies on the flow rate and the standard curve. However, the standard curves were plotted according to the relationship between retention time and peak molecular weight (*M*_p_). This means that the determination of *M*_w_ depended on the calculation of measured *M*_p_. Since the molecular mass distribution of dextran was uneven during the degradation, *M*_p_ can hardly match *M*_w_. This might cause great errors in the measurement. Moreover, it is also worth mentioning that in most of the previous studies, the molecular mass distribution of dextrans was not measured, despite that this parameter is important in that it directly reflects the quality of dextran. The polydispersity index, which is defined as the ratio of weight-average molecular weight and number-average molecular weight (*M*_w_/*M*_n_), is an index to describe the molecular mass distribution. 

In this work, a novel dextranase was purified from *Penicillium cyclopium* CICC-4022 and its properties were characterized. The effects of temperature, pH and metal ions on dextranase activity were evaluated. The dextranase was then applied to degrade dextran. We found that *M*_w_ of the degraded dextrans could be effectively controlled by adjusting the reaction time, concentrations of the dextranase and the substrate concentration during the degradation. *M*_w_ and *M*_w_/*M*_n_ of the degraded dextrans was analysed using gel permeation chromatography (GPC) coupled with multiple-angle laser light scattering (GPC-MALS).

## 2. Results and Discussions

### 2.1. Purification of Dextranase

**Figure 1:**
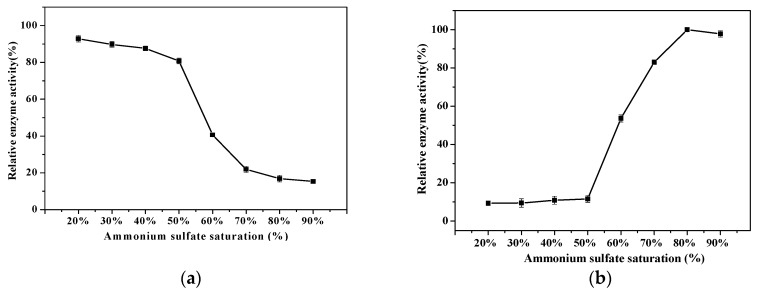
Relative dextranase activity of the supernatant (**a**) and the precipitate (**b**) with different ammonium sulphate saturation.

It can be seen from Figure 1, when ammonium sulfate saturation was less than 50%, the relative dextranase activity of the supernatant was above 80.73% (taking the dextranase activity of the crude enzyme as 100%) and that of the precipitate was no more than 11.50% (taking the highest dextranase activity as 100%), only a small amount of the dextranase and miscellaneous proteins were salted out and most of the enzyme was in the supernatant. When ammonium sulfate saturation was 80%, the relative dextranase activity of the supernatant was 16.89%. In this latter case, most of the dextranase has been precipitated and the relative activity of the precipitate was high. To increase the recovery rate of the dextranase and not bring extra miscellaneous proteins during the process, the precipitate of 50–80% saturation should be collected. Dextranase was purified to 1.71-fold concentration and its yield was 87.35%. The specific activity of the purified enzyme was 111.06 U/mg (Table 1).

**Figure 2:**
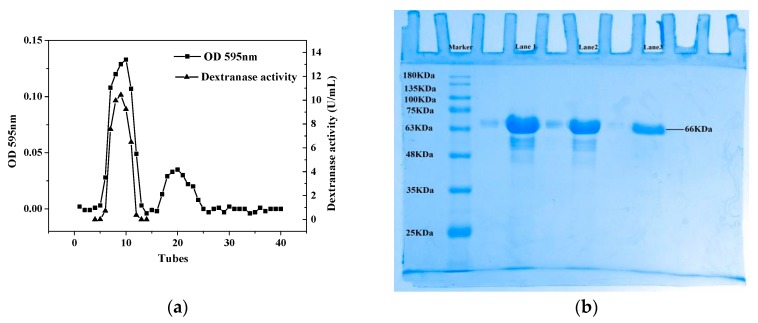
(**a**) Gel filtration chromatography results of the dextranase on Sepharose 6B; (**b**) SDS-PAGE results of the dextranase. For (**b**), lane markers represent marker proteins; Lane 1: the crude enzyme; Lane 2: the ammonium sulphate precipitate; Lane 3: the purified dextranase, collected from gel filtration chromatography.

It can be seen from Figure 2a that the dextranase has been effectively eluted and separated using Sepharose 6B chromatography. Fractions with high specific activity were collected from Tube 4 to 14. Dextranase was purified to 16.09-fold concentration and its yield was 29.17%. The specific activity of the purified enzyme reached 350.29 U/mg (Table 1). 

The samples from Tube 7, 8, 9 and 10 were merged and analysed by SDS-PAGE. As shown in Figure 2b, only one band appeared in Lane 3, suggesting the dextranase from *Penicillium cyclopium* was a monomer and the purification process was effective. *M*_w_ of purified dextranase was about 66 kDa, which was similar to dextranases from other fungi such as *Aspergillus allahabadii* X26 (66 kDa) [12] and *Talaromyces pinophilus* H6 (58kDa) [13] as reported previously. 

### 2.2. Dextranase Enzymatic Properties

#### 2.2.1. Effect of Temperature on Dextranase Activity and Thermal Stability

**Figure 3:**
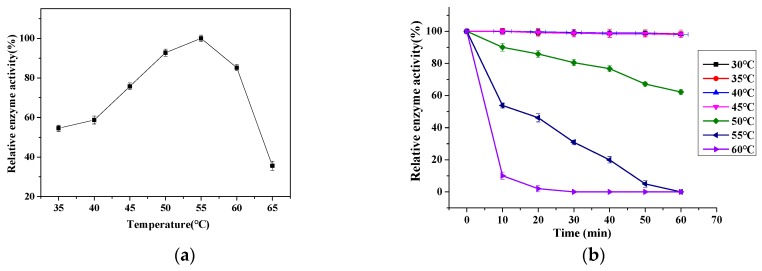
Relative enzymatic activity of the dextranase as a function of temperature (**a**) and time (**b**).

Figure 3a shows the relative enzyme activity of the dextranase as affected by temperature. With increasing temperature, the relative activity firstly increased and then decreased. When temperature varying from 35 to 60 °C, the relative activity was always higher than 54.55%; and the highest relative activity was reached at 55 °C. It is worth mentioning that this optimal temperature of 55 °C was higher than those dextranase from *Hypocrea lixii* F1002 [4] (25 °C) and *Bacillus* sp [22] (50 °C) reported previously. This demonstrates the higher thermal stability of our dextranase from *Penicillium cyclopium* than that of dextranase from other sources.

Figure 3b shows the changes in relative enzymatic activity of the dextranase stored at pH of 5 from 45 °C to 60 °C for 10–60 min. After stored at 45 °C and 50 °C for 60 min, the dextranase activity remained relatively stable with the values being 98.09% and 62.21%, respectively. This suggests that the dextranase from *Penicillium cyclopium* had higher thermal stability than those from *Catenovulum* sp DP03 [15] and *Arthrobacter oxydans* [23].

#### 2.2.2. Effect of pH on Dextranase Activity and pH Stability

**Figure 4:**
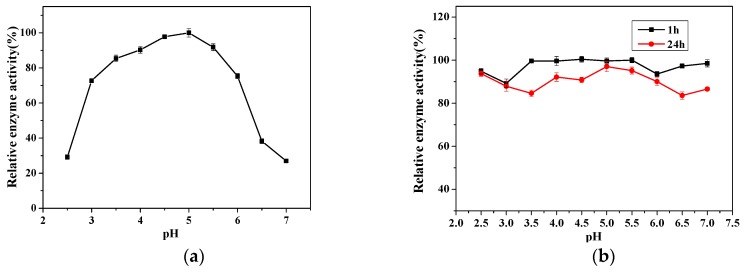
Relative enzymatic activity of the dextranase as a function of pH (**a**) and as a function of pH for 1 h and 24 h (**b**).

Figure 4a shows the relative enzyme activity at different pH values. With increasing pH, the relative activity firstly increased and then decreased and the highest relative activity was achieved at a pH of 5. With pH ranged from 3.0 to 6.0, the relative enzyme activity was higher than 72.67%. It is worth noting that with pH of 3.0, the relative enzyme activity of our dextranase from *Penicillium cyclopium* was more than 72.67%, while the dextranase from *Talaromyces pinophilus* H6 [13] was only 45%. This indicates that our dextranase has better acid-resistance compared to other dextranases obtained from *Penicillium*.

Figure 4b shows the changes in the relative enzymatic activity of dextranase stored at a pH of 2.5–7 for 1 h and 24 h, respectively. It can be seen that the relative activity for both storage times was stable. The relative activity was more than 89.22% after 1 h and more than 84.58% after 24 h. This performance was better than the dextranase from *Chaetomium globosum* [24]. Therefore, our dextranase from *Penicillium cyclopium* has greater acid-resistance and suitable to be applied in a wide pH range.

#### 2.2.3. Substrate Specificity and Final Products Analysis of Enzyme

Dextranase activity during the catalysed hydrolysis of substrates with diverse glucosidic bonds was measured to evaluate the substrate specificity of the dextranase (Table 2). The substrates containing α-1,6 glucosidic bonds had higher relative dextranase activity. This means that our dextranase specifically degraded the α-1,6 glucosidic bonds of dextran and the specificity is similar to dextranases from other sources [4,25,26]. With an increase in dextran *M*_w_, the relative enzyme activity firstly increased and then decreased and dextran T70 was the optimum substrate for the dextranase. The dextranase was less effective for soluble starch, which contains most α-1,4 glucosidic linkages and limited amounts of a-1,6 linkages [2,27]. Moreover, the dextranase showed no effects on cellulose, sucrose, chitin, chitosan and β-cyclodextrin. Thus, our dextranase showed high specificity toward dextrans that contain mostly α-1,6 glucosidic bond and it was inactive with the α-1,4, β-1,2, β-1,4 glucosidic bonds of the substrate.

**Figure 5:**
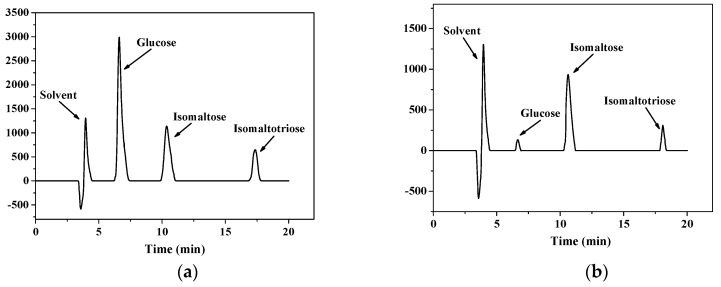
HPLC results for glucose, isomaltose and isomaltotriose (**a**); and for the final products of dextranase (**b**).

HPLC results show that the peaks of glucose, isomaltose and isomaltotriose appeared at 6.6, 10.4 and 17.4 min, respectively (Figure 5a). The peaks of our final products of dextranase were consistent with the peaks of glucose, isomaltose and isomaltotriose (Figure 5a). It is known that the final products of endodextranase were mainly isomaltose, isomaltotriose and a small amount of glucose [2]. Therefore, it can be concluded that the dextranase from *Penicillium cyclopium* was an endodextranase.

#### 2.2.4. Effects of Metal Ions and Compounds on Enzyme Activity

The effects of several metal ions and compounds on dextranase activity are listed in Table 3. The results show that Li^+^, Na^+^, Fe^2+^ and Tris could improve the dextranase activity at all concentrations, especially in 0.001 M Li^+^, where the activity could reach 116.28%. In addition, NH^4+^, Tris and urea slightly enhanced the enzymolysis. Fe^3+^, Ca^2+^ and EDTA slightly reduced the dextranase activity [13]. Regarding SDS, low concentrations (0.001 M) had positive effects on dextranase activity, while high concentrations had negative effects. Cu^2+^, Zn^2+^, Cr^2+^, Pb^2+^ and Ni^2+^ all inhibited the enzymolysis. Furthermore, with increasing concentration, this inhibition effect became stronger. The dextranase activity was only 16.41% when Cu^2+^ was 0.01 M.

#### 2.2.5. Kinetic Parameters of Dextranase

The *K*_m_, *V*_m_, *K*_cat_, *K*_cat_/*K*_m_ values of dextran T5, T70 and T2000 are listed in Table 4. *K*_m_ is a parameter to evaluate the affinity with the substrate; higher *K*_m_ indicates lower affinity. It can be seen that with an increase in *M*_w_ of the substrate, *K*_m_ decreased, indicating stronger affinity of the dextranase to the dextran with high *M*_w_; The *K*_m_ value of our dextranase for dextran T2000 was 2.45 mM, which was smaller than those of dextranases from *Arthrobacter oxydans* (4.73 mM) [28], *Aspergillus allahabadii* X26 (14.29 mM) [12] and *Streptomyces* sp. NK458 (94.30 mM) [29]. This shows the stronger affinity of the dextranase from *Penicillium cyclopium* than that of the dextranases reported previously.

The *Kcat* and *K*_cat_/*K*_m_ value of the dextranase for dextran increased with increasing *Mw* of the dextran, which showed that the dextranase from Penicillium cyclopium has higher catalytic efficiency for high-*Mw* dextran.

### 2.3. Determination of M_w_ and M_w_/M_n_ of Dextran

#### 2.3.1. Standard Curve of Dextran of GPC

**Figure 6:**
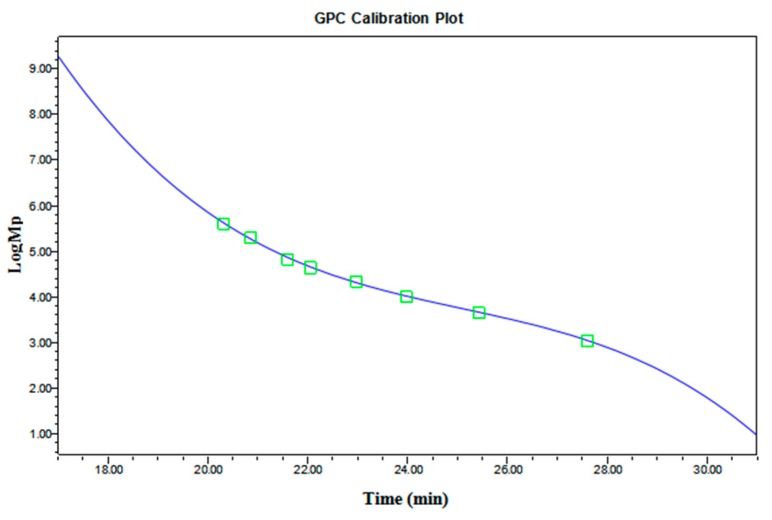
The standard curve of dextran.

GPC is the most commonly used technique for determining the molecular mass of polymers including dextran. Using this technique, the relative *M*_w_ value of dextran can be obtained based on the standard curve of dextran (Figure 6). According to Section 3.7.1, the retention time was taken as the abscissa, the logarithmic values of *M*_p_ were taken as the ordinate and the standard curve is obtained by fitting using Waters Breeze 2. The standard curve of dextran was calculated to be: log(*M*_p_) = *s* − 1.31*t* + 0.512*t*^2^ − 0.0068*t*^3^, *R*^2^ = 0.9993, *σ* = 2.88%. The third-order function fits well with minimal error.

#### 2.3.2. Comparison of GPC-MALS with GPC and Error Analysis

**Figure 7:**
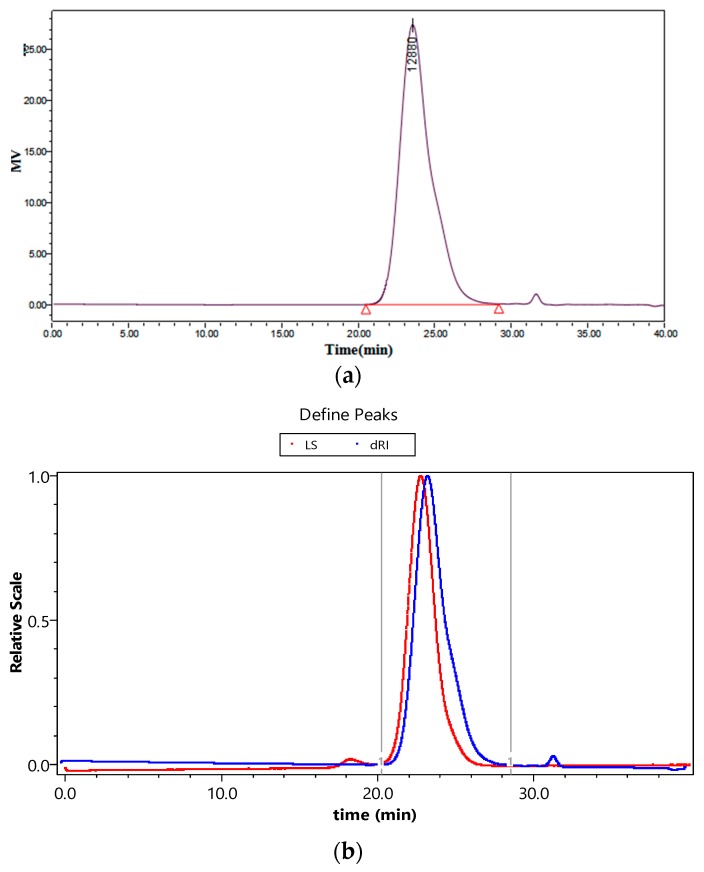
Chromatograms of GPC (**a**) and GPC-MALS (**b**).

Figure 7 presents the chromatograms of GPC (a) and GPC-MALS (b), both of which show a well-defined peak. For both techniques, the sample peaks display a symmetrical normal distribution. Figure 7a has a signal from the refractive index detector and *M*_p_, *M*_w_ and *M*_w_/*M*_n_ were calculated using Breeze 2 software (Waters) based on the standard curves. Figure 7b has two signal peaks from the refractive index detector and MALS respectively and the curves were generated by ASTRA 7.1.3 software of MALS (Wyatt). The differential signal should be used for the occurrence of the peak while the laser signal should be taken for the conclusion of the peak. In this way, we measured *M*_p_, *M*_n_, *M*_z_, *M*_w_ and *M*_w_/*M*_n_ of dextrans.

Table 5 shows the values of *M*_w_ and *M*_w_/*M*_n_ and relative errors of *M*_w_ and *M*_w_/*M*_n_ of dextrans measured by GPC and GPC-MALS. The relative errors of *M*_w_ of dextran measured by GPC-MALS were less than 7%, while those measured by GPC were more than 10% and even up to 40%. The relative errors of *M*_w_/*M*_n_ of dextran measured by GPC-MALS were less than 8%, while the maximum relative error measured by GPC was 160.7%. Thus, compared to GPC, GPC-MALS produced much smaller relative errors of *M*_w_ and *M*_w_/*M*_n_ of dextrans. The reason could be that the relative *M*_w_ values measured by GPC mainly depends on the standard curve and pump speed. Moreover, *M*_w_ must be within the linear range of the standard curve; for generating the standard curve, the standard samples with larger *M*_w_ (>1000 kDa) was difficult to obtain and store. On the contrary, these factors are not related to GPC-MALS, which could be considered as a more accurate method to measure *M*_w_ and *M*_w_/*M*_n_ of dextrans.

#### 2.3.3. Degradation of Dextran Polymers by Dextranase

The changes in *M*_w_ and *M*_n_/*M*_w_ of dextran with time under different enzyme activity and substrate concentrations are shown in Table 6 and Table 7, respectively. It can be seen that *M*_W_ decreased rapidly during the first 4 min and did slowly, suggesting that the enzyme had higher affinity with higher-*M*_W_ dextrans [24]. When *M*_W_ was 100–1000 kDa, *M*_n_/*M*_w_ was higher, which might be contributed by dextran molecules of diverse *M*_w_ in the system. With the reaction proceeded, *M*_w_ decreased and gradually reached to a plateau, and, thus, *M*_n_/*M*_w_ became smaller. For example, when the enzyme activity was 0.2 U/mL, *M*_w_ and *M*_n_/*M*_w_ of the dextran after 8 min of hydrolysis were 720.6 kDa and 4.524, respectively, which decreased to 51.16 kDa and 1.966 respectively after 30 min.

Dextranase activity and dextran concentration are two important factors that can affect the degradation rate. Higher dextranase activity could lead to an increased degradation rate of dextrans. As listed in Table 6, when the dextranase activity was increased from 0.2 U/mL to 0.6 U/mL, *M*_W_ of dextran after 8 min of hydrolysis decreased from 720.6 kDa to 83.69 kDa. On the other hand, increasing dextran concentration could decrease the degradation rate. As listed in Table 7, when the dextran concentration was increased from 1% to 5%, *M*_W_ of dextran after 16 min of hydrolysis increased from 7.196 kDa to 63.9 kDa. Based on these results, we can conclude that *M*_W_ of dextran can be effectively controlled by adjusting the dextranase activity, dextran concentration and reaction time [24,30].

## 3. Materials and Methods

### 3.1. Materials

Dextrans T3, T5, T20, T40, T70, T500 and T2000 (*M*_w_ ≈ 3, 5, 20, 40, 70, 500 and 2000 kDa) and a series of standards (dextran 1.27, 5.52, 11.6, 23.8, 48.6, 80.9, 273.0, 667.8 kDa) were purchased from Sigma-Aldrich (St. Louis, MO, USA). Sepharose 6B was a product from Henghui (Beijing, China). The protein marker was obtained from ShineGene Molecular Biotech (Shanghai, China). *Penicillium cyclopium* (CICC-4022) was purchased from the China Centre of Industrial Culture Collection (Beijing, China). All other chemicals were of analytical grade and purchased from Sinopharm Chemical Reagent Co., Ltd. (Beijing, China).

### 3.2. Preparation of Crude Dextranase

According to our preliminary experiments with dextran, sucrose, glucose, maltose and fructose for optimized carbon sources, we have assumed that that α-1,6 glycosidic bonds in dextran played an inducing role in the production of dextranase. A Czapek Dox medium was used as the solid medium and seed culture medium for *Penicillium cyclopium*. The fermentation medium was prepared by dextran T20 30 g/L, yeast extract 4 g/L, KCl:FeSO_4_·7H_2_O = 10:1 (concentration ratio) and K_2_HPO_4_ 1.0 g/L. The mixture had an initial pH of 6.1. 60 mL of the fermentation medium was transferred into a 250mL flask, which was then sterilized at 121 °C for 30 min. Inoculum concentration was set as 3% and the fermentation medium was incubated in a rotary shaker at 160 r/min and 30 °C for 72 h. The thalli of *Penicillium cyclopium* were separated by centrifugation for 20 min at 4 °C and 10,000× *g*. The supernatant was stored at 4 °C for further dextranase purification. 

### 3.3. Dextranase Activity Assay

Dextranase activity was assayed by the determination of the amount of substance of reducing sugar from Dextran T70. The diluted dextranase of 1.0 mL was incubated with 1.0 mL 3% dextran T70 (preparation with 0.02 M acetate buffer of pH 5) at 50 °C for 10 min, the amount of reducing sugar was obtained using the 3,5-dinitrosalicylic acid method as reported previously [31]. One unit (1 U) of dextranase activity was defined as the amount of enzyme that degrades dextran T70 to produce 1 µmol glucose equivalent per min at 50 °C and pH of 5 [32] and calculated according to Equation (1).

Dextranase activity (U/mL) = [Amount of reducing sugar (µmol) × Dilution multiple of enzyme solution]/[Volume of enzyme solution (mL) × Time (min)](1)

### 3.4. Determination of Protein Concentration

Protein concentration (mg/mL) was measured by the Bradford method [33]. Crystalline bovine serum albumin was used as the protein standard. 

### 3.5. Purification of Dextranase

The cell-free filtrate (CFF) was subjected to ammonium sulfate fractionation with a saturation of 20–90%. The precipitate was collected by centrifugation at 10,000× *g* for 20 min at 4 °C. Then the precipitate was dissolved and dialyzed in 0.02 M acetate buffer (pH 5.0) overnight at 4 °C. The concentrated dextranase was loaded on a Sepharose 6B column (1.5 cm i.d. × 50 cm) and the dextranase was eluted with 0.02 M acetate buffer (pH 5.0) at a flow rate of 0.2 mL/min. The absorbance of each fraction (3 mL) was determined at 595 nm to monitor the proteins during chromatographic separation. The fractions with high dextranase activity were gathered to measure their dextranase activity and protein concentration as described in Section 3.3 and Section 3.4. Specific activity was calculated according to the above-measured results. The purity of the dextranase and its *M*_w_ were determined by sodium dodecyl sulfate polyacrylamide gel electrophoresis (SDS-PAGE). The unstained protein marker was used as control samples, *M*_w_ of the dextranase was analysed by a 5% concentrated gel and a 12% separated gel. Following electrophoresis, the gel was stained with Coomassie blue R-250 and decolorized several times with a destainer.

### 3.6. Dextranase Enzymatic Properties

#### 3.6.1. Effect of Temperature and pH on Dextranase Activity and Stability

For investigating the effect of temperature, dextran T70 (30 mg/mL) and the dextranase were prepared with 0.02M acetate buffer (pH = 5.0). The effect of temperature on dextranase activity was measured at 35–65 °C. Relative activity was expressed as percentage of the highest activity while the highest dextranase activity is 100%. The thermal stability of dextranase was measured by exposing the dextranase in 0.02M acetate buffer (pH = 5.0) for 10–60 min at 45–65 °C without any substrate. 

For studying the effect of pH, dextranase activity was determined at pH of 2.5–7.0 at 55 °C. Relative activity was expressed as percentage of the highest activity while the highest dextranase activity was 100%. The pH stability of the dextranase was evaluated by incubating the dextranase in reaction buffers at pH 2.5–7.5 at 4 °C for 1 h and 24 h respectively without any substrate.

For both thermal and pH stability, dextran T70 was added to the dextranase to measure the relative dextranase activity. The activity of non-heated and non-stored dextranase was taken as 100% dextranase activity, respectively. 

#### 3.6.2. Substrate Specificity and Analysis of Hydrolysis Products

Dextran T3, T5, T20, T40, T70, T500 and T2000, soluble starch, cellulose, sucrose, chitin, chitosan and β-cyclodextrin (30 mg/mL) were prepared with 0.02M acetate buffer (pH = 5.0) and the dextranase activity was measured with these different substrates. Relative activity was expressed as percentage of the highest activity so that the highest dextranase activity is 100%.

The final hydrolysis products of dextran T70 were analysed by high-performance liquid chromatography (HPLC) with a mobile phase of 80% acetonitrile. Glucose, isomaltose and isomaltotriose were used as the standards. The hydrolysates were diluted to 10-fold with the mobile phase and then centrifuged at 11,000× *g* for 20 min. The supernatant was filtered with a 0.22 μm filter and analysed by HPLC, in which a Polaris NH_2_ (4.6 mm × 250 nm, Agilent Technologies, Santa Clara, CA, USA) column was used. The chromatograph was operated under a flow rate of 1.0 mL/min at 35 °C and then connected to an RID-10A refractive index detector (Shimadzu Corp. Kyoto, Japan).

#### 3.6.3. Effects of Metal ions and other Compounds on Dextranase Activity

Metal ions and reagents (namely, Cu^2+^ (CuSO_4_), Fe^3+^ (FeCl_3_), Zn^2+^ (ZnSO_4_), Ca^2+^ (CaCl_2_), Co^2+^ (CoCl_2_), K^+^ (KCl), Li^+^ (LiCl), Na^+^ (NaCl), Pb^2+^ (Pb(NO_3_)_2_), Ni^2+^ (NiCl_2_), NH_4_^+^ (NH_4_Cl), Fe^2+^ (FeSO_4_), Tris, SDS, urea and EDTA) were dissolved with 0.02 M acetate buffer (pH = 5.0). The concentrations of these solutions were 0.001, 0.005 and 0.01 M, respectively. The effects of different ions and reagents on dextranase activity were measured. Dextranase activity without any compounds was considered as 100% to calculate the relative activity of the above compounds. 

#### 3.6.4. Enzyme Kinetics

In order to determine the kinetic constants, the initial velocity (*v*) was measured with various concentrations (0.1–0.6%) of Dextran T5, T70 and T2000 in 0.02M acetate buffer (pH = 5.0) at 55 °C for 6 min. The kinetic constants were calculated from Lineweaver–Burk plots [34].

### 3.7. Determination of M_w_ and M_w_/M_n_

#### 3.7.1. Gel permeation chromatography (GPC)

The chromatographic conditions used were as follows: the mobile phases: 0.03% NaN_3_ and 0.1 M NaNO_3_; the columns: Ultrahydrogel^TM^ 2000 (7.8 mm × 300 mm, Shimadzu, Japan), Ultrahydrogel^TM^ 250 (7.8 mm × 300 mm, Shimadzu, Japan) and Ultrahydrogel^TM^ DP120A (7.8 mm × 300 mm, Shimadzu, Japan); flow rate: 1 mL/min; the detector: Waters 2414 refractive index detector (Waters Corporation, Milford, MA, USA); column temperature: 35 °C; and detector temperature: 35°C.

The dextran standards with *M*_w_ of 1.27, 5.52, 11.6, 23.8, 48.6, 80.9, 273.0 and 667.8 kDa (*M*_p_ = 1.08, 4.44, 9.89, 21.4, 43.5, 66.7, 196.3 and 401.3 kDa, respectively) were prepared with a concentration of 1 mg/mL using the mobile phase. The samples were injected after filtration with a 0.22 μm water filter, GPC was performed to detect the retention time for different *M*_w_. Waters Breeze 2 software was used to generate the standard curve of dextran.

Both GPC and GPC-MALS techniques were used to determine *M*_w_ and *M*_w_/*M*_n_ of dextran. The relative errors of *M*_w_ and *M*_w_/*M*_n_ determined by the two techniques were analysed and compared. Except that GPC-MALS had a refractive index detector and a laser detector, the two techniques used the same chromatographic conditions.

#### 3.7.2. Degradation of dextrans by dextranase

GPC-MALS was used to measure *M*_w_ and *M*_w_/*M*_n_ of dextrans. *M*_w_, *M*_n_ and *M*_w_/*M*_n_ can be obtained without depending on the pump speed and the standard curve. Pawcenis et al. [35] have used this method to measure *M*_w_ of cellulose. 

Dextran T2000 (1%, 3%, 5%) and *Penicillium cyclopium* dextranase (dextranase activity: 0.2, 0.4 and 0.6 U/mL) were prepared with 0.02 M acetate buffer (pH = 5.0). The dextranase was incubated with the substrate at 50 °C. Samples were obtained at intervals, boiled for at least 3 min to stop the reaction and diluted to 10 folds with the mobile phase. The samples were filtered with a 0.22 μm water filter. *M*_w_ and *M*_w_/*M*_n_ of dextran were analysed by GPC-MALS (Waters Corporation, Milford, MA, USA; Wyatt Technology Corporation, Santa Barbara, CA, USA) according to the conditions stated in Section 3.7.1.

## 4. Conclusions

In this work, we obtained a novel dextranase from *Penicillium cyclopium* CICC-4022. After purification to 16.09-fold concentration, the dextranase showed specific enzymatic activity of 3780.13 U/mg and molecular weight of 66 kDa. For this dextranase, the optimum temperature and pH were 55 °C and 5.0, respectively. The dextranase was stable below 45 °C under pH from 3.5 to 7. We found that this dextranase could specifically degrade α-1,6 glucosidic bonds of dextran and could be identified as an endodextranase. Besides, some metal ions such as Li^+^, Na^+^ and Fe^2+^ could effectively promote the dextranase activity. The obtained dextranases were then applied to degrade dextrans with excellent performance. Moreover, we found *M*_w_ of dextran could be effectively controlled by adjusting the dextranase activity, substrate concentration and reaction time during degradation. Therefore, our findings show the high potential of this dextranase from *Penicillium cyclopium* CICC-4022, which can be potentially applied to the production of specific dextrans for clinical applications.

## Figures and Tables

**Table 1:** Parameters related to purification of the dextranase *from Penicillium cyclopium*.

Purification Step	Total Protein (mg)	Total Activity (U)	Specific Activity (U/mg)	Purification (Fold)	Yield (%)
Culture dextranase	7.08	533.15	75.32	1.00	100.00
(NH4)_2_SO_4_ precipitation	4.19	465.71	111.06	1.71	87.35
Sepharose 6B	0.44	155.54	350.29	16.09	29.17

**Table 2:** Effects of dextranase on diverse carbohydrates.

Substrate	Main Linkage	Relative Activity (%)
DextranT3	α-1,6	65.17 ± 0.7
DextranT5	α-1,6	65.54 ± 0.6
DextranT20	α-1,6	90.64 ± 0.4
DextranT40	α-1,6	98.50 ± 0.2
DextranT70	α-1,6	100.00 ± 0.5
DextranT500	α-1,6	99.62 ± 0.1
DextranT2000	α-1,6	97.00 ± 0.4
Soluble starch	α-1,4; α-1,6	20.83 ± 0.8
Cellulose	β-1,4	0
Sucrose	β-1,2	0
Chitin	β-1,4	0
Chitosan	β-1,4	0
β-Cyclodextrin	β-1,4	0

**Table 3:** Effects of metal ions and compounds on the activity of dextranase.

Compound	Relative Activity (%)	Relative Activity (%)	Relative Activity (%)
	(0.001 M)	(0.005 M)	(0.01 M)
Control	100	100	100
Cu^2+^	24.02 ± 0.15	22.53 ± 0.05	16.41 ± 0.2
Fe^3+^	99.55 ± 0.10	64.61 ± 0.07	67.27 ± 0.07
Zn^2+^	90.95 ± 0.02	59.35 ± 0.13	16.85 ± 0.05
Ca^2+^	101.81 ± 0.03	93.30 ± 0.04	40.73 ± 0.06
Cr^2+^	90.95 ± 0.08	72.74 ± 0.12	48.25 ± 0.06
K^+^	97.74 ± 0.06	114.83 ± 0.10	105.75 ± 0.05
Li^+^	116.28 ± 0.05	115.78 ± 0.09	103.98 ± 0.12
Na^+^	104.98 ± 0.07	111.48 ± 0.02	105.75 ± 0.05
Pb^2+^	59.29 ± 0.05	22.53 ± 0.03	20.39 ± 0.15
Ni^2+^	95.48 ± 0.15	97.61 ± 0.05	67.71 ± 0.16
NH_4_^+^	107.58 ± 0.04	105.21 ± 0.07	99.53 ± 0.15
Fe^2+^	104.74 ± 0.06	100.95 ± 0.02	109.95 ± 0.12
Tris	103.79 ± 0.08	100.00 ± 0.07	100.47 ± 0.10
SDS	106.16 ± 0.03	46.94 ± 0.05	34.15 ± 0.13
Urea	112.79 ± 0.04	109.00 ± 0.11	99.05 ± 0.04
EDTA	102.37 ± 0.07	80.58 ± 0.14	81.05 ± 0.05

**Table 4:** Kinetic constants of purified dextranase from *Penicillium cyclopium.*

	Dextran T5 ^b^	Dextran T70 ^b^	Dextran T2000 ^b^
*K*_m_ (mM)	2.72 ± 0.04	2.61 ± 0.02	2.45 ± 0.01
*V*_max_ (µmol _Glucose_ min^−1^ mg^−1^_Protein_)	189.50 ± 0.03	192.70 ± 0.04	203.21 ± 0.01
*K*^a^_cat_ (s^−1^)	208.45 ± 0.01	211.97 ± 0.02	223.53 ± 0.03
*K*_cat_/*K*_m_ (M^−1^ s^−1^)	76636.0 ± 0.06	81214.6 ± 0.03	91236.7 ± 0.05

^a^*K*_cat_ value was calculated assuming a molecular mass of 66 kDa for native dextranase. ^b^ The results reported were the means of three replications ± SD.

**Table 5:** Error analysis of GPC and GPC-MALS.

Standard	*M*_w_ (kDa)		*M*_w_ δ (%)		*M*_w_/*M*_n_		*M*_w_/*M*_n_ δ (%)	
	GPC	GPC-MALS	GPC	GPC-MALS	GPC	GPC-MALS	GPC	GPC-MALS
Dextran (*M*_w_ ≈ 1.27 kDa)	0.84	1.32	−33.86	3.94	1.72	1.20	36.51	−4.76
Dextran (*M*_w_ ≈ 5.52 kDa)	3.09	5.4	−44.02	−2.17	3.03	1.57	89.38	−1.88
Dextran (*M*_w_ ≈ 23.8 kDa)	21.4	23.99	−10.08	0.80	1.28	1.229	24.62	7.69
Dextran (*M*_w_ ≈ 48.6 kDa)	31.57	49.98	−35.04	2.84	1.28	1.24	−5.88	−5.15
Dextran (*M*_w_ ≈ 80.9 kDa)	59.26	82.26	−26.74	1.68	1.64	1.28	12.33	−5.48
Dextran (*M*_w_ ≈ 667.8 kDa)	827.80	621.5	23.96	−6.93	5.24	2.049	160.70	1.94

**Table 6:** *M*_w_ and *M*_n_/*M*_w_ of dextran under different dextranase activity and time.

Reaction Time (min)	0.2 U/mL		0.4 U/mL		0.6 U/mL	
	*M*_w_ (kDa)	*M*_n_/*M*_w_	*M*_w_ (kDa)	*M*_n_/*M*_w_	*M*_w_ (kDa)	*M*_n_/*M*_w_
0	1869	3.236	1859	1.523	1859	3.358
4	1553	2.322	865.9	3.048	251	5.891
8	720.6	4.524	242.3	4.272	83.69	3.991
12	426.5	5.329	69.25	3.12	48.34	2.198
16	216	4.837	40.74	2.504	24.13	2.133
20	119.5	4.373	29.54	2.071	20.78	1.917
30	51.16	1.966	16.77	1.82	11.6	1.731
40	32.41	1.618	11.35	1.672	9.247	1.507
50	22.26	2.187	9.685	1.542	7.156	1.502
60	13.79	1.627	8.052	1.433	6.262	1.36
90	11.39	1.494	6.362	1.306		
120	8.239	1.375	5.954	1.238		
150	7.358	1.396				

**Table 7:** *M*_w_ and *M*_n_/*M*_w_ of dextran under different substrate concentrations and time.

Reaction Time (min)	Dextran (1%)		Dextran (3%)		Dextran (5%)	
	*M*_w_ (kDa)	*M*_n_/*M*_w_	*M*_w_ (kDa)	*M*_n_/*M*_w_	*M*_w_ (kDa)	*M*_n_/*M*_w_
0	1726	2.995	1759	3.358	1750	3.829
4	49.94	2.534	251	5.891	487.8	11.383
8	16.06	1.702	83.69	3.991	194.4	6.03
12	10.65	1.39	48.34	2.198	107.1	5.032
16	7.196	1.388	24.13	2.133	63.9	3.519
20	5.893	1335	20.78	1.917	42.4	2.964
30	4.993	1.234	11.6	1.731	23.88	2.237
40	4.434	1.53	9.247	1.507	16.54	1.979
50			7.156	1.502	12.8	1.693
60			6.262	1.36	10.05	1.651
